# Navigating the transition of care in patients with inborn errors of immunity: a single-center’s descriptive experience

**DOI:** 10.3389/fimmu.2023.1263349

**Published:** 2023-10-03

**Authors:** María Alejandra Mejía González, Patricia Quijada Morales, María Ángeles Escobar, Alba Juárez Guerrero, María Elena Seoane-Reula

**Affiliations:** ^1^ Immunology Department, Hospital General Universitario Gregorio Marañón, Madrid, Spain; ^2^ Primary Immunodeficiencies Unit (National Reference Center for Primary Immunodeficiencies (PID)), Hospital General Universitario Gregorio Marañón, Madrid, Spain; ^3^ Pediatric Immuno-Allergy, Allergy Department, Hospital General Universitario Gregorio Marañón, Madrid, Spain; ^4^ Day-care Hospital of Immunology, Department of Nursing of Day-care Hospital, Hospital General Universitario Gregorio Marañón, Madrid, Spain; ^5^ Medical Advisor of the Spanish Association of Primary Immunodeficiencies (AEDIP), Madrid, Spain

**Keywords:** transition of care, primary immunodeficiencies, complex chronic conditions, combined immunodeficiencies with syndromic features, inborn errors of immunity

## Abstract

The transition from pediatric to adult care is a critical milestone in managing children, especially in those with complex chronic conditions. It involves ensuring the patient and family adapt correctly to the new phase, maintaining continuity of ongoing treatments, and establishing an appropriate follow-up plan with specialists. Patients with Inborn error of immunity (IEI), formerly known as Primary Immune Disorders (PID) are part of a group of disorders characterized by alterations in the proper functioning of the immune system; as the diagnostic and treatment tools for these entities progress, life expectancy increases, and new needs emerge. These children have special needs during the transition. Particularly important in the group of children with PID and syndromic features, who often present multiple chronic medical conditions. In these cases, transition planning is a significant challenge, involving not only the patients and their families but also a wide range of specialists. To achieve this, a multidisciplinary transition team should be established between the pediatric specialists and the adult consultants, designing a circuit in which communication is essential. As few transition care guidelines in the field of PID are available, and to our knowledge, there is no specific information available regarding patients with PID associated with syndromic features, we share our experience in this issue as a Primary Immunodeficiencies Unit that is a National Reference Center for PID, and propose a guide to achieve an adequate and successful transition to adulthood in these patients, especially in those with associated syndromic features.

## 1 Introduction

Transition of Care (TOC) is the process of moving from a child/family-centered model of care to an adult/patient-centered model of care (1). Planning that transition is fundamental to make it less stressful and more successful for both, parents and children.

Transition to adulthood is recognized as a complicated process for youth in general and more so in those with special health care needs (2, 3). While moving toward adulthood, adolescents with special health care needs must learn how to self-manage their medical needs as well as face an imminent shift in service location. For patients with Inborn error of immunity (IEI), formerly known as PID, the transition requires more in-depth planning and ongoing support because a variety of specialists are involved in their care.

Published studies continue to reveal the adverse effects associated with a lack of structured TOC interventions in terms of medical complications (4), limitations in health and wellbeing (5), problems with treatment and medication adherence (6), discontinuity of care, patient dissatisfaction, higher emergency department and hospital use (7), and higher costs of care. Considering this, transition of care guidelines have been made, but specific guides to transition pediatric immunodeficiency patients are lacking.

According to the results of a survey applied to members of the American Academy of Allergy Asthma and Immunology (AAAAI) and Clinical Immunology Society (CIS), the transition of care of PID patients remains overlooked in our specialty and providers want and need additional training and resources, 24.1% of respondents did not have a TOC policy and only 25.0% were satisfied with their current practices (8).To these difficulties adds up the lack of availability of transition of care or even the access to an immunologist in some countries (9). Therefore, there is a clear need to develop and evaluate the effectiveness of evidence-based guidelines, resources, and best practices for PID patients.

This paper aims to describe how PIDs uniquely impact the process of transition preparation, the challenges they pose for the transfer of care and future directions based in our experience.

Our Unit is a designated National Reference Center by the Ministry of Health in clinical management of PID in Spain, accumulating over 30 years of expertise in the field. It has a strong culture of multidisciplinary work, and regularly attends to complex patients. We will attempt to provide new insights to understand the intricacies of transitioning from pediatric to adult care settings in PID by focusing on children with PID and syndromic features.

## 2 Inborn errors of immunity as complex chronic conditions

The IEI are a heterogeneous group of over 480 disorders characterized by poor or absent function in components of the innate and/or adaptive immune systems. The majority of the more severe IEI are manifest in childhood and used to have an ominous prognosis in terms of morbidity and mortality; however, the improvement in their diagnosis and management has resulted in a modification of the natural history of the disease and therefore has brought as consequence, in an increased survival of pediatric patients with IEI, who now can be considered as chronic patients and need transfer to adult services for life-long follow-up (10–12).

In addition, each IEI group presents different characteristics, so they are patients with great fragility, multimorbidity and complexity in the transition process.

The model of care for patients with IEI with syndromic features should be similar to that of patients with Complex Chronic Conditions (CCC) requiring urgent care in the outpatient setting to treat acute health problems, having at least one outpatient provider who comprehensively addresses acute and chronic medical, functional, and psychosocial needs (13); and requiring coordination of decision-making among all health care providers, developing effective care plans to maximize the child’s well-being which should include a transition plan (14, 15).

**Figure 1:**
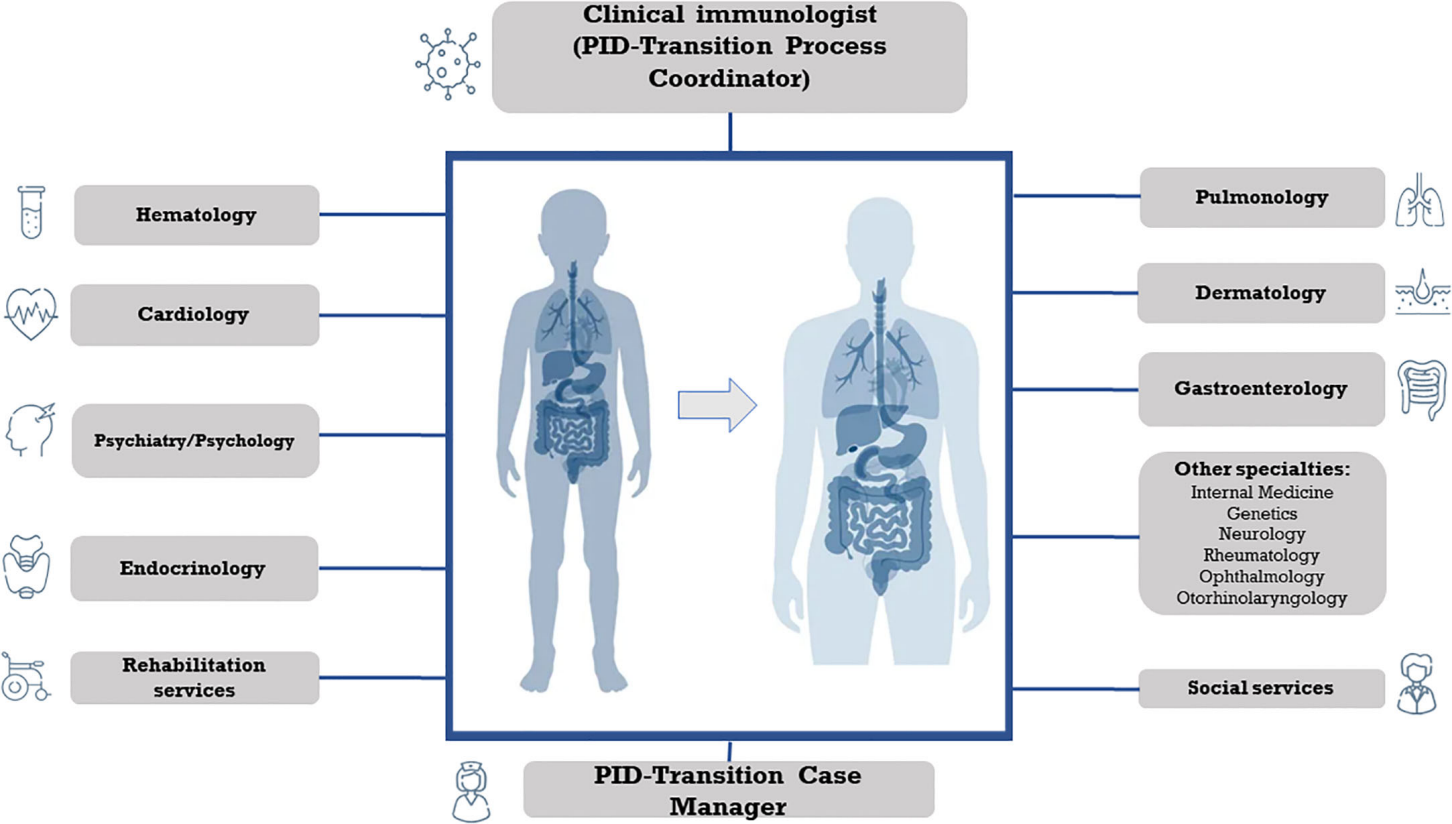
Multidisciplinary approach in the transition of care of Primary Immunodeficiency with syndromic features.

The fact that patients with IEI are more susceptible to severe and/or recurrent infections, autoimmune processes, allergies, and certain types of neoplasms implies that many specialists are involved in their care; this takes on a special relevance in patients with syndromic IEI, who present complicated pathologies and comorbidities. Nowadays, the transition of care has evolved from a focus on pediatric care responsibility to a shared responsibility by pediatrics and adult care clinicians (physicians, nurses, social workers). The pediatric immunologist can act as a leader in the coordination of the other specialists that participate in the patient’s care (**Figure 1**).

## 3 Complexity in the transition of care process

The challenges that per se face adolescents and young adults living with IEI are amplified by the need for a transition from pediatric to adult health care.

Adolescence is a critical period, particularly for adolescents and young adults (from 10 up to 24 years) with a chronic medical condition, being increasingly recognized as a vulnerable population (16, 17). In addition to their underlying illness, they may confront situations such as adaptive difficulties, mental health issues, school-related problems, and be involved in hazardous behaviors such as substance abuse and exposition to sexually transmitted diseases.

While moving toward adulthood, adolescents with CCC must learn about their own illness and how to self-manage their medical needs as well as face the up-coming shift in service location (the actual transfer). The need to take on responsibility for behaviors and learn to cope with everyday events and challenges falls increasingly on the adolescent.

Among the transition challenges that are intrinsic to the adolescence period, we can find the physical, moral, emotional, and psychological changes that condition the lack of decision-making skills, and self-care skills in this population (1, 18, 19). On the one hand, they demand more independence, but on the other hand, they cannot take responsibility for taking medications and keeping appointments because they have been dependent on their parents or guardians throughout their childhood.

The natural desire to become more independent can be redirected so that they accept the likely change of location and healthcare personnel that will attend to them in adulthood and reinforce that continuity in care will minimize morbidity and mortality. The relationship between the multidisciplinary pediatric and adult team is essential to achieve this.

Therapeutic adherence is another important challenge during transition in IEI. For many chronically treated patients, antibiotic prophylaxis, immunoglobulin replacement, chronic immunosuppressants, or biologic therapy are the cornerstone of their therapy and need to be administered at fixed intervals. Lack of treatment adherence has been demonstrated as a pitfall in the TOC of other chronic medical conditions (6, 7, 19). Consequently, the strategies focused on the education of patients regarding their treatment are a crucial point of empowerment for young adults and, in this aspect, is of particular importance the role of the pediatric and adult’s Advanced Nurse Specialist in Immunology. It has been reported that the nurses’ intervention in transition, targeting parents and young adults, will improve parents’ readiness for their child’s transfer to adult health care and strengthen the adolescent’s self-management skills (20, 21).

It is important to note that there are an increasing number of patients with IEI who have undergone hematopoietic stem cell transplantation (HSCT) and gene therapy. All these individuals require long-term follow-up and transfer to an adult clinic with HSCT and gene therapy expertise should be considered (11). The care of these patients demands units that possess extensive experience and exceptional specialization in IEI.

Lastly, we must consider the difficulties inherent in the system, such as: lack of communication, coordination, and transfer of medical records between adult and pediatric clinician or system. In this regard, group meetings, including doctor, nursing staff, social workers, and psychologists from both pediatric and adult departments, should be held to help complete the missing aspects in the patient’s history when necessary.

## 4 Proposed care

The transition process of patients with IEI should be individualized, staged, and gradual. The appropriate time for transition is determined by the patient’s psychological maturity and readiness, as chronological age does not necessarily correspond to the official age for transition. Due to the administrative difficulties involved, the transition period has been established from 16 to 18 years old, although, for example, a delay in neurocognitive development due to IEI with syndromic features, or a severe episode of infection or malignancy would advise delaying the transition.

We recommend being flexible in the age at which transfer to PID Adult Units takes place, considering the mental age of development and the patient’s and family’s opinions, without significant delay in the transfer. It should be discussed from the age of 12, and thus it will be internalized as an issue that will come.

Healthcare personnel should plan before puberty and set a date that the adolescent will know approximately 1 year in advance, offering written material and giving time to ask questions. At about age 14 years, the pediatric immunologist will start to conduct part of the clinic visits without the parents present to help the patient to feel more comfortable and independent with health care choices. However, this may not be always possible, and the patient’s cognitive capacity must be considered in order to deliver care with the highest level of respect and consideration.

For parents, the transition means a loss of control in caring for their child, and they are often reluctant to lose this responsibility. They should understand the need to hand over responsibility and acquire the role of providers of support. The idea that the transition to adulthood is the achievement of a goal should be reinforced. One of the most effective ways to facilitate the process is to encourage them to attend meetings organized by nursing staff for health education with groups of young adults with IEI who have already gone through this transition.

As described before, transitional planning should be a coordinated, multidisciplinary, interactive and collaborative process. At our site, we have two pivotal figures that lead the PID-TOC process: the Transition Coordinator and the Transition Case Manager.

**Figure 2:**
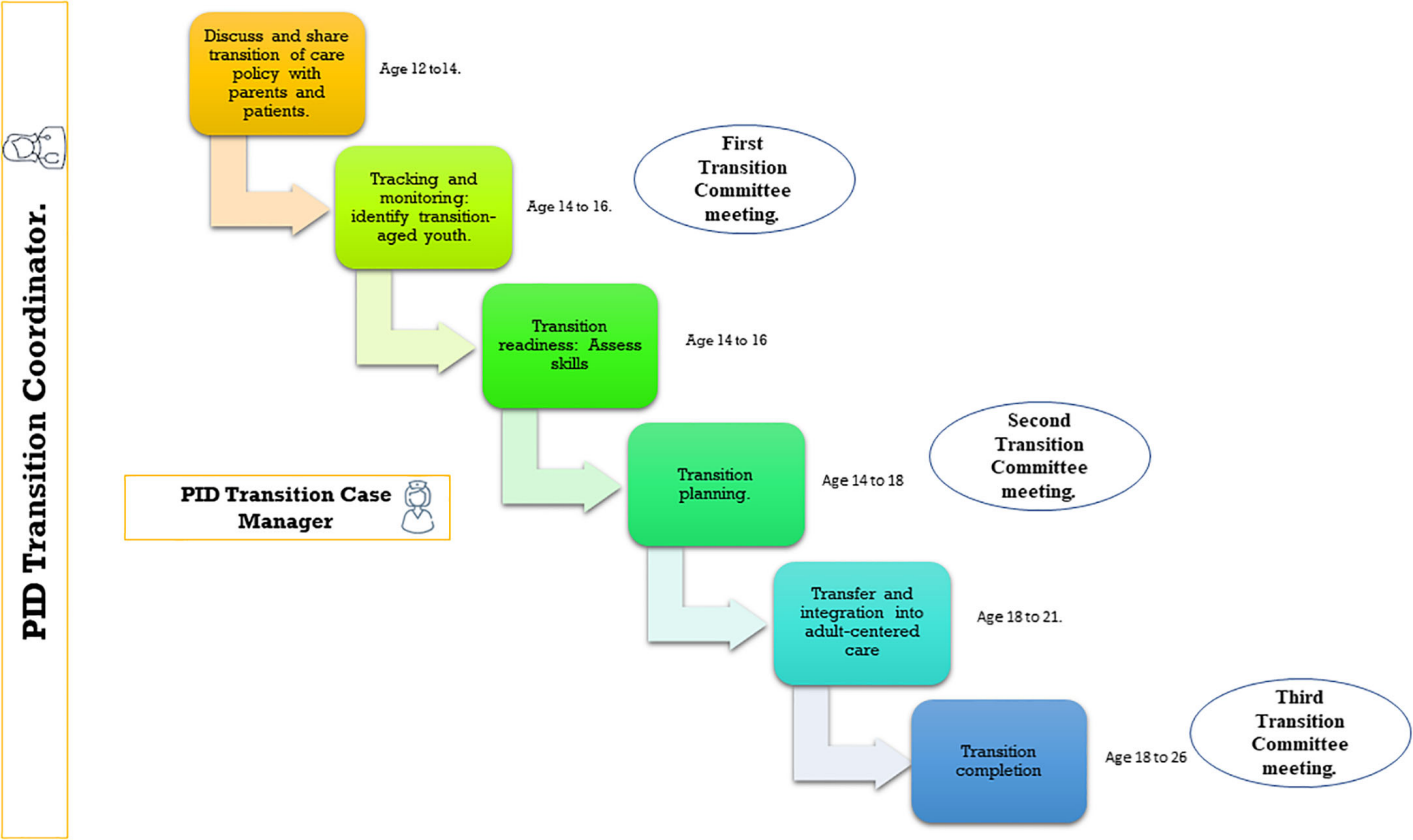
Overview of the application of the Six Core Elements for the Transition Process. Adapted from: White et al. Supporting the Health Care Transition From Adolescence to Adulthood in the Medical Home. *Pediatrics*. 2018;142(5):e20182587.

The Transition Coordinator is the pediatric immunologist who has overseen the patient since the diagnosis and now has the role of organizing the care of the adolescent by different healthcare personnel who will be involved in the care when entering adulthood. The Transition Coordinator supervises that the transition’s six core elements proposed by White P. *et*, tailored for PID (**Figure 2**) (22). These steps include establishing a transition and care policy, tracking and monitoring the transition, assessing transition readiness, planning the transition, initiating transfer of care and the transfer completion (22) and organizes a set of three multidisciplinary meetings in what we call the PID Transition Committee (TC) (**Figure 2**). The PID-TC will be form by all the professionals involved in the care of the child with IEI, as well as the adult specialists responsible for their care in adulthood.

These meetings take place in different times:

- First meeting PID-TC: the case is presented to all the specialists that are involved in the care of the patient when he is14-year-old.
- Second meeting PID-TC: this is an intermediate meeting to supervise how the transition process is going and to address any problems or difficulties.
- Third meeting PID-TC: takes place once the TOC is completed when the patient is approximately 18 years old.

Although all healthcare professionals are present at PID-TC meetings, for youth with multiple specialists involved in their care, we recommend sequential rather than simultaneous transition to different adult physicians.

The PID Transition Case Manager figure is assumed by an Advance Practice Nurse. The case manager is present from the beginning of the transition of care process but usually begins meeting with patients at age 16 or 17 and subsequently at their routine outpatient visits, which are generally scheduled every 6 months, or during their visits to the Day-care Hospital if the patient receives active treatment. At these meetings, the Transition Case Manager discusses how patients are progressing toward self-managed care and concerns about the transfer, introduces the patient and his family to the adult team, and shows them the facilities in the adult clinics. Lastly, the Case Manager supervises the Step by step check list adapted for PID (**Supplementary Material**).

At our hospital, there is continuity between the pediatric and adult services as all the staff belongs to the Primary Immunodeficiency Unit. We hold biweekly meetings to discuss complex cases, monitor the clinical progress of patients, and explore new diagnostic and therapeutic approaches. This allows the Unit’s professionals to provide comprehensive and individualized medical care to each patient, which greatly facilitates the transition process.

The transition process should be accompanied by protocols for the care of PIDs in the Emergency Department, to address the needs that may arise in this area.

When assessing the perception of quality of care in our Unit: 75% of the patients reported being satisfied and 25% very satisfied. These data cover the quality of care in consultations, Day Clinic, and the TOC protocol.

### 4.1 Transition of care in syndromic PIDs

Our Unit has amassed a vast repertoire of experience in managing patients diagnosed with PID accompanied by various complex syndromic features. Our expertise extends to a wide range of conditions, such as Kabuki syndrome, DiGeorge syndrome, Hyper-IgE syndrome, and many others.

Recognizing the significance of this transitional phase, we have developed a comprehensive approach to address the specific needs of these patients, prioritizing open communication, information sharing, and continuity of care to facilitate a smooth transfer of responsibilities between healthcare providers.

The patients with syndromic IEI are the quintessential complex chronic patient. They present with developmental disorders, sensory problems, malformations, and multiple organ dysfunction (23). Complex syndromic patients often require multidisciplinary medical care and management, as their needs can be complex and varied. The improvements in the diagnosis and treatment of these patients have led to an increase in life expectancy and, therefore an increase in the number of patients to be transferred to adult units, so that their management frequently requires the collaboration of several medical specialties to comprehensively address the patient’s multiple conditions (**Figure 1**) (24).

In addition to the complexity of managing multiple organ involvement, there is added difficulty, given the fact that most of them show some kind of intellectual disability, with different levels of cognitive impairment, and other comorbidities such as autism spectrum disorder or anxiety (25, 26). Based on this, they should receive appropriate care from a psychiatry/psychology specialist who can ease the transition.

It is known that adolescents with development disabilities appear to be less encouraged to take responsibility for their care, but the decision-making capacity should be assessed to establish a plan (27, 28). These patients face a variety of difficulties, affecting their daily activities, having problems in the social area with less daily activities and isolation because of anxiety and stress and impaired verbal communication skills. The unemployment rate and the lack of proper education is also a significant handicap (29). When the patient is not capable of its own care, is the parent or caregiver who has the responsibility to proceed with it, and this should be contemplated in the TOC to adult plan.

As the clinical presentation of IEI changes over the course of life, the need for evaluation by different specialties will also vary. During infancy the priority is the organ malformations, as the patient grows, they should be referred to a specialist who would help them develop communication skills, give them mental health tools and resources to improve the behavioral and educational aspects (30).

It is important that in the period as they mature into adulthood, adolescents with IEI receive sexual health education, including reproductive health care, as well as genetic counselling about the possibility of having affected offspring (31).

We must not forget that in all patients with IEI, especially in those with intellectual disabilities, the burden of this disease affects the family unit as a whole, not only affecting the parents or the direct caregivers, but additionally it may take a toll on the siblings who are at risk of developing emotional and behavioral difficulties (32); for this reason, they should be included as part of the care plan (33). Even though a multidisciplinary approach ought to be stablished, the impact of frequent medical appointments and therapy sessions should be taken into account when planning the transition (34).

In our experience the transition plan should consider the adolescents and their families, which need to be actively included on behalf of the patients, who cannot always make their own decisions.

## 5 Conclusions

Establishing a successful transition for patients with IEI, especially in those with multiple chronic conditions as the syndromic population, requires a comprehensive approach in which the multidisciplinary collaboration, the patient and family integration, and the effective team coordination is essential to complete a successful transition.

The multidisciplinary team comprises various healthcare professionals, with the PID-Transition Process Coordinator and the PID-Transition Case Manager as cornerstones of the transition, providing not only the immunological care but also coordinating the psychosocial, educational, and developmental needs of the patient ensuring a smooth transition and working closely with the multidisciplinary team to develop an individualized transition plan, monitor progress, and address any challenges that may arise.

The patient and family integration are crucial during the process as they need to be actively involved in the decision-making, goal setting, and care planning, maintaining an open communication and education to ensure an adequate understanding of the PID transition process and the empowerment to participate actively. It is important to remark that in some patients, complex illness and intellectual disability have a unique impact on the transition readiness process, and pose specific challenges for the transfer of care to the adult.

Through our extensive experience and ongoing dedication, we are deeply committed to facilitating a successful transition into adulthood for patients with IEI, especially complex syndromic patients. Our goal is to ensure continuity of care, promote overall well-being and improve the quality of life of these individuals as they navigate the challenges associated with their conditions.

We present a proposed working methodology based on our experience in the transition process of primary immunodeficiencies. We hope that this proposal proves useful as a starting point for achieving a successful transition to adulthood for the most challenging patients, as it poses a significant healthcare challenge.

## Data availability statement

The original contributions presented in the study are included in the article/**Supplementary Material**. Further inquiries can be directed to the corresponding author.

## Author contributions

MM: Conceptualization, Investigation, Methodology, Writing – original draft. PQ: Conceptualization, Investigation, Writing – original draft. ME: Writing – review & editing. AJ: Writing – original draft. MS-R: Conceptualization, Investigation, Project administration, Supervision, Writing – review & editing.

## References

[1] White PH Cooley WC. Transitions clinical report authoring group; American Academy of Pediatrics; American Academy of Family Physicians; American College of Physicians. Supporting the health care transition from adolescence to adulthood in the medical home. Pediatrics (2018) 142(5):e20182587. doi: 10.1542/peds.2018-258 30348754

[2] Ishizaki Y Matsuo M Saito K Fujihira Y. Factors surrounding the healthcare transition from pediatric to adult care in 5p- syndrome: A survey among healthcare professionals. Front Pediatr (2022) 10:924343. doi: 10.3389/fped.2022.924343 35874599 PMC9304764

[3] Sakurai I Maru M Miyamae, T Honda M. Prevalence and barriers to health care transition for adolescent patients with childhood-onset chronic diseases across Japan: A nation-wide cross-sectional survey. Front Pediatrics (2014) 10:956227. doi: 10.3389/fped.2022.956227 PMC9476551 36120652

[4] Wafa S Nakhla M. Improving the transition from pediatric to adult diabetes healthcare: A literature review. Can J Diabetes (2015) 39:520–8. doi: 10.1016/j.jcjd.2015.08.003 26498219

[5] Chaudhry SR Keaton M Nasr SZ. Evaluation of a cystic fibrosis transition program from pediatric to adult care. Pediatr Pulmonol (2013) 48:658–65. doi: 10.1002/ppul.22647 22888094

[6] Annunziato RA Baisley MC Arrato N Barton C Henderling F Arnon R. Strangers headed to a strange land a pilot study of using a transition coordinator to improve transfer from pediatric to adult services. J Pediatr (2013) 163:1628–33. doi: 10.1016/j.jpeds.2013.07.031 23993138

[7] Shaw KL Southwood TR McDonagh JE. Young people’s satisfaction of transitional care in adolescent rheumatology in the UK. Child Care Health Dev (2007) 33:368–79. doi: 10.1111/j.1365-2214.2006.00698.x 17584391

[8] Rai S Treyster Z Jongco AM. Knowledge, attitudes, and practices of allergists/immunologists regarding transition of care for primary immunodeficiency patients. J Clin Immunol (2023) 43:595–603. doi: 10.1007/s10875-022-01415-1 36454452

[9] Nordin J Solís L Prévot J Mahlaoui N Chapel H Sánchez-Ramón S. The PID principles of care: where are we now? A global status report based on the PID life index. Front Immunol (2021) 12:780140. doi: 10.3389/fimmu.2021.780140 34868053 PMC8637458

[10] Guffroy A Martin T Korganow AS. Adolescents and young adults (AYAs) affected by chronic immunological disease: A tool-box for success during the transition to adult care. Clin Immunol (2018) 197:198–204. doi: 10.1016/j.clim.2018.10.010 30347239

[11] Mahlaoui N Warnatz K Jones A Workman S Cant A. Advances in the care of primary immunodeficiencies (PIDs): from birth to adulthood. J Clin Immunol (2017) 37:452–60. doi: 10.1007/s10875-017-0401-y PMC5489581 28523402

[12] Israni M Nicholson B Mahlaoui N Obici L Rossi-Semerano L Lachmann H. Current transition practice for primary immunodeficiencies and autoinflammatory diseases in europe: a RITA-ERN survey. J Clin Immunol (2023) 43:206–16. doi: 10.1007/s10875-022-01345-y PMC9840587 36222999

[13] Browning CJ Thomas SA. Implementing chronic disease self-management approaches in Australia and the United Kingdom. Front Public Health (2015) 2:162. doi: 10.3389/fpubh.2014.00162 25964902 PMC4410761

[14] Cohen E Berry JG Camacho X Anderson G Wodchis W Guttmann A. Patterns and costs of health care use of children with medical complexity. Pediatrics (2012) 130(6):e1463-70. doi: 10.1542/peds.2012-0175 23184117 PMC4528341

[15] Tyack Z. The greatest challenges and solutions to improve children’s health and well-being worldwide in the next decade and beyond: Using complex systems and implementation science approaches. Front Pediatr (2023) 11:1128642. doi: 10.3389/fped.2023.1128642 36923277 PMC10009164

[16] Vazquez-Ortiz M Gore C Alviani C Angier E Blumchen K Comberiati P. A practical toolbox for the effective transition of adolescents and young adults with asthma and allergies: An EAACI position paper. Allergy: Eur J Allergy Clin Immunol (2023) 78:20–46. doi: 10.1111/all.15533 PMC10091987 36176045

[17] Society T. Young adult health and well-being: A position statement of the society for adolescent health and medicine. J Adolesc Health (2017) 60:758–9. doi: 10.1016/j.jadohealth.2017.03.021 28532650

[18] Sable C Foster E Uzark K Bjornsen K Canobbio MM Connolly HM. Best practices in managing transition to adulthood for adolescents with congenital heart disease: The transition process and medical and psychosocial issues: A Scientific Statement from the American Heart Association. Circulation (2011) 123:1454–85. doi: 10.1161/CIR.0b013e3182107c56 21357825

[19] Schraeder K Allemang B Scott C McBrien K Dimitropoulos G Felske A. Primary care during the transition to adult care for adolescents involved with pediatric specialty services: a scoping review protocol. Syst Rev (2021) 10(1):46. doi: 10.1186/s13643-021-01593-w 33531077 PMC7856752

[20] Joo JY Liu MF. The experience of chronic illness transitional care: A qualitative systematic review. Clin Nurs Res (2022) 31:163–73. doi: 10.1177/10547738211056166 34727782

[21] Thomsen EL Boisen KA Hanghøj S Hansson H Grabow Scheelhardt HCV Christensen ST. A comprehensive transfer program from pediatrics to adult care for parents of adolescents with chronic illness (ParTNerSTEPs): study protocol for a randomized controlled trial. Trials (2022) 23:1–12. doi: 10.1186/s13063-022-06997-0 36539857 PMC9768961

[22] White P Schmidt A Shorr J Ilango S Beck D McManus M. Six Core Elements of Health Care Transition^TM^ 3.0. Washington, DC: Got Transition, The National Alliance to Advance Adolescent Health (2020).

[23] Cirillo A Lioncino M Maratea A Passariello A Fusco A Fratta F. Clinical manifestations of 22q11.2 deletion syndrome. Heart Fail Clin (2022) 18:155–64. doi: 10.1016/j.hfc.2021.07.009 34776076

[24] Cirillo E Giardino G Ricci S Moschese V Lougaris V Conti F. Consensus of the Italian Primary Immunodeficiency Network on transition management from pediatric to adult care in patients affected with childhood-onset inborn errors of immunity. J Allergy Clin Immunol (2020) 146:967–83. doi: 10.1016/j.jaci.2020.08.010 32827505

[25] Caciolo C Alfieri P Piccini G Digilio MC Lepri FR Tartaglia M. Neurobehavioral features in individuals with Kabuki syndrome. Mol Genet Genom Med (2018) 6:322–31. doi: 10.1002/mgg3.348 PMC6014453 29536651

[26] Kalinousky AJ Rapp T Hijazi H Johnson J Bjornsson HT Harris JR. Neurobehavioral phenotype of Kabuki syndrome: Anxiety is a common feature. Front Genet (2022) 13:1007046. doi: 10.3389/fgene.2022.1007046 36276984 PMC9582441

[27] Nugent J Gorman G Erdie-Lalena CR. Disparities in access to healthcare transition services for adolescents with down syndrome. J Pediatr (2018) 197:214–20. doi: 10.1016/j.jpeds.2018.01.072 29571933

[28] Varshney K Iriowen R Morrell K Pillay P Fossi A Stephens MM. Disparities and outcomes of patients living with Down Syndrome undergoing healthcare transitions from pediatric to adult care: A scoping review. Am J Med Genet A (2022) 188:2293–302. doi: 10.1002/ajmg.a.62854 PMC9545419 35686676

[29] Cortés-Martín J Peñuela NL Sánchez-García JC Montiel-Troya M Díaz-Rodríguez L Rodríguez-Blanque R. Deletion syndrome 22q11.2: A systematic review. Children (Basel) (2022) 9(8):1168. doi: 10.3390/children9081168 36010058 PMC9406687

[30] Habel A Herriot R Kumararatne D Allgrove J Baker K Baxendale H. Towards a safety net for management of 22q11.2 deletion syndrome: Guidelines for our times. Eur J Pediatr (2014) 173:757–65. doi: 10.1007/s00431-013-2240-z PMC4032642 24384789

[31] Fung WLA Butcher NJ Costain G Andrade DM Boot E Chow EWC. Practical guidelines for managing adults with 22q11.2 deletion syndrome. Genet Med (2015) 17:599–609. doi: 10.1038/gim.2014.175 25569435 PMC4526275

[32] Smith S Tallon M Clark C Jones L Mörelius E. “You never exhale fully because you’re not sure what’s NEXT”: parents’ Experiences of stress caring for children with chronic conditions. Front Pediatr (2022) 10:902655. doi: 10.3389/fped.2022.902655 35832577 PMC9271768

[33] Quintana Mariñez MG Chakkera M Ravi N Ramaraju R Vats A Nair AR. The other sibling: a systematic review of the mental health effects on a healthy sibling of a child with a chronic disease. Cureus (2022) 14(9):e29042. doi: 10.7759/cureus.29042 36249634 PMC9550208

[34] Theodore-Oklota C Egan S Paulich M Evans CJ Hartman DS Hoffman DL. Caregiver-reported clinical characteristics and the burden associated with Kabuki syndrome. Am J Med Genet A (2020) 182:1592–600. doi: 10.1002/ajmg.a.61584 PMC7383624 32246746

