# Supplementary material for: Navigating the transition of care in patients with inborn errors of immunity: a single-center’s descriptive experience
Source: Front Immunol. 2023 Oct 3;14:1263349. doi: 10.3389/fimmu.2023.1263349 (PMC10579936; doi:10.3389/fimmu.2023.1263349)
Supplement: Supplementary file 1 [file DataSheet_1.pdf]

<table>
<tr><th colspan="5">Step-by-step transition care plan in Primary Immunodeficiencies</th></tr>
<tr><td>Name</td><td colspan="4">Date of birth</td></tr>
<tr><td colspan="5">Address</td></tr>
<tr><td colspan="5">Legal guardian name</td></tr>
<tr><td>Primary immunodeficiency diagnosis</td><td colspan="4">Other diagnoses</td></tr>
<tr><td colspan="5">Pediatric immunologist</td></tr>
<tr><td colspan="5">Pediatric nurse</td></tr>
<tr><td colspan="2">Transition care plan proposed and discussed with the patient and caregiver</td><td>Date</td><td>Date</td><td>Date</td></tr>
<tr><td colspan="2">Transition readiness assessment</td><td>Date</td><td>Date</td><td>Date</td></tr>
<tr><td colspan="2">Adult transition team notification</td><td>Date</td><td>Date</td><td>Date</td></tr>
<tr><td colspan="2">Transition team meeting</td><td>Date</td><td>Date</td><td>Date</td></tr>
<tr><td colspan="2">Summarized medical history and emergency plan of action</td><td>Date</td><td>Date</td><td>Date</td></tr>
<tr><td colspan="2">Revised and updated plan of care</td><td>Date</td><td>Date</td><td>Date</td></tr>
<tr><td colspan="5">Designated adult immunologist</td></tr>
<tr><td>Name</td><td colspan="4">Contact information</td></tr>
<tr><td>Notification of patient transition</td><td colspan="4">Date</td></tr>
<tr><td>First appointment</td><td colspan="4">Date</td></tr>
<tr><td colspan="5">Designated adult nurse</td></tr>
<tr><td>Name</td><td colspan="4">Contact information</td></tr>
<tr><td>First appointment</td><td colspan="4">Date</td></tr>
<tr><td colspan="5">Transfer package:</td></tr>
<tr><td colspan="5">◻ Transfer letter</td></tr>
<tr><td colspan="5">◻ Transition plan of care</td></tr>
<tr><td colspan="5">◻ Last transition readiness assessment</td></tr>
<tr><td colspan="5">◻ Medical summary (Disease course, past treatment, co-morbidities and complications including infections, autoimmunity, allergy, cancer history, etc.)</td></tr>
<tr><td colspan="5">◻ Current plan of care, including ongoing treatment protocol (e.g., immunoglobulin replacement therapy schedule, immunosuppressant or biologic therapy, etc.)</td></tr>
<tr><td colspan="5">◻ Emergency action plan (What to do in case of an emergency, recommended tests, etc.)</td></tr>
<tr><td colspan="5">◻ Condition fact sheet</td></tr>
<tr><td colspan="5">◻ Guardianship and other pertaining legal documents</td></tr>
<tr><td colspan="5"></td></tr>
<tr><td>◻ Prepared</td><td colspan="4">Date</td></tr>
<tr><td>◻ Sent</td><td colspan="4">Date</td></tr>
</table>

Step-by-step transition care plan in primary immunodeficiencies. Adapted from: White et al. Supporting the Health Care Transition From Adolescence to Adulthood in the Medical Home. *Pediatrics.* 2018;142(5):e20182587.
